# From blood coagulation to innate and adaptive immunity: the role of platelets in the physiology and pathology of autoimmune disorders

**DOI:** 10.1007/s00296-018-4001-9

**Published:** 2018-02-28

**Authors:** Zuzanna Małgorzata Łukasik, Marcin Makowski, Joanna Samanta Makowska

**Affiliations:** 1 Department of Rheumatology, Medical University of Lodz, Ul. Pieniny 30, 92-115 Łódź, Poland; 2 Department of Intensive Care, Cardiology, Medical University of Lodz, Łódź, Poland

**Keywords:** Blood platelets, Autoimmunity, Rheumatoid arthritis, Systemic lupus erythematosus, Systemic sclerosis, Anti-phospholipid antibody syndrome

## Abstract

Thrombosis and cardiovascular complications are common manifestations of a variety of pathological conditions, including infections and chronic inflammatory diseases. Hence, there is great interest in determining the hitherto unforeseen immune role of the main blood coagulation executor—the platelet. Platelets store and release a plethora of immunoactive molecules, generate microparticles, and interact with cells classically belonging to the immune system. The observed effects of platelet involvement in immune processes, especially in autoimmune diseases, are conflicting—from inciting inflammation to mediating its resolution. An in-depth understanding of the role of platelets in inflammation and immunity could open new therapeutic pathways for patients with autoimmune disorders. This review aims to summarize the current knowledge on the role of platelets in the patomechanisms of autoimmune disorders and suggests directions for future research.

## Introduction

Platelets are anucleate derivatives of the megakaryocytic cytoplasm. They were long perceived as participating solely in haemostasis and thrombosis [1]. As many as 100 billions of these discoid, cell elements with a relatively short life span need to be produced daily to maintain the average platelet count of 2–3 × 10^8^ per blood ml [2]. Platelets are equipped with megakaryocyte clusters of differentiation, various surface receptors and glycoproteins, cytoskeletal elements, granules, and a smooth endoplasmic reticulum tubular system [3]. These properties, together with platelets’ ubiquitous presence, make them perfect candidates for immune cells. Currently, platelets are gaining increasing recognition as active regulators of innate and adaptive immunity [4]. With chronic systemic inflammation being regarded as the axis of the pathogenesis of autoimmune disorders (AD) and elevated risk of cardiovascular events among AD patients, research into the contribution of platelets in the pathogenesis and course of these diseases is particularly attractive [5, 6]. The aim of this comprehensive narrative review is to present the most current evidence for platelet involvement as immune cells in the course of systemic AD, discuss usefulness of platelet indices in clinical practice, and point towards future areas of research.

## Literature search and review methodology

Literature searches were conducted between April and September 2017 using MEDLINE database, comprised of published data only and included both existing review articles and original studies in English. The search used the following search string: ‘blood platelets’ AND ‘autoimmunity’ OR ‘autoimmune diseases’ OR ‘immune’. Preference was given to the sources published since 2002. Articles and abstracts were screened for relevance and only those covering systemic autoimmune disorders were included. References in the articles meeting inclusion criteria were examined as well.

## Platelets as immune cells

### The role of platelets in haemostasis

Blood flow forces platelets along the vessel wall, where they act as sentinels of vascular integrity. Disruption of the vessel wall results in exposure of extracellular matrix components such as subendothelial collagen, vitronectin, fibronectin, or laminin. Platelet glycoprotein complex GP Ib/IX/V and various surface integrins work as initial adhesive receptors and enable platelet binding at the site of an endothelial lesion. This binding initiates the adhesion, activation, and accumulation of platelets, which together is regarded as the first step of haemostasis. Platelets also contribute to the second step: the blood coagulation pathway [7]. Upon platelet activation, changes in the composition of the phospholipid bilayer of the plasma membrane occur. Unsaturated acyl chains are exposed on the outer leaflet. The platelet cell membrane bears a nett negative charge, and this accelerates the activation of factor X and prothrombin, by providing sites for the assembly of enzyme substrate complexes. Changes in the platelet cell membrane lead also to the generation of platelet-derived microparticles and a body of evidence points towards their role as initiators of thrombosis via the tissue factor pathway [8]. In mouse models, platelets were shown to become hyperactive after induction by microparticles derived from damaged endothelial cells [9]. This represents a positive feedback loop further enhancing the already increased coagulant activity of the platelets and putting them in the centre of thrombotic events.

However, a variety of pathological conditions, which are not strictly associated with vascular damage, manifest as a tendency towards thrombosis or as excessive bleeding [10]. This includes conditions as diverse as bacterial and parasite infections, malignant neoplasms, and autoimmune disorders. This observation has inspired a new route of research, investigating whether platelets should be regarded as immune cells [11].

### Platelets’ surface receptors

An examination of platelet surface reveals it to be bristling with receptors. Quiescent platelets bear receptors intended to efficiently monitor vascular integrity. Upon activation, platelets upregulate other types of receptors, and shared with classic constituents of the immune system [12]. For example, toll-like receptors (TLRs) enable platelets to recognize pathogen- and damage-associated molecular patterns and immune complexes. TLRs also enable numerous interactions between platelets and leukocytes [12]. Ligand binging results in the initiation of intracellular signalling that converges upon significant cytoskeletal changes, involving the formation of pseudopodia and the opening of the canalicular system. These changes provide small-sized cells with a greater surface area, enable release of stored molecules, generation of microvesicles and microparticles and, most importantly, rearrangement of surface proteins [13]. Siglec receptors are involved in platelet apoptosis and down-regulation of the inflammatory response [14–20].

Two of the platelet surface proteins should be discussed more thoroughly due to their role in predicting cardiovascular complications. Glycoprotein integrin αIIbβ3 (GPIIb/IIIa) is the most abundant platelet receptor with 50–80 thousand copies on the cell surface and an additional pool stored in the α granules [19]. An increase in the intracellular level of calcium leads to conformational changes of the molecule and subunit association, which only jointly form a functional complex. The surface density of the complex increases as well. This enables the cross-linking of plasma fibrinogen or von Willebrand Factor (vWF) and consequent platelet aggregation and intracellular communication, necessary for the recruitment of additional platelets to the site of vascular injury and thrombus formation. Platelet glycoprotein Ib alpha chain (GPIba), a component of the Glycoprotein Ib-IX-V receptor complex, is also up-regulated; it facilitates further platelet adhesion to the endothelium and supports intercellular signalling, also with neutrophils [21, 22]. P-selectin is present only on activated platelets (but also expressed on endothelial cells) and, therefore, serves as a marker of platelet activation. The counter ligand for P-selectin (PSGL-1) is a homodimeric mucin expressed on most leukocytes, but especially abundant on monocytes and neutrophils. The interaction is crucial for adaptive immunity as it induces leukocyte activation, integrin Mac-1 surface clustering (which enables other adhesion interactions between platelets and leukocytes), and neutrophil transendothelial migration. In addition, P-selectin enables the binding of complement C3b.

### Platelets’ granules

Together with the changes occurring in the composition and quantity of their surface proteins, activated platelets also release the contents of their granules. Platelets contain almost 4000 unique proteins, more than 300 of which have been detected in platelet releasates. Many of them are megakaryocyte-preformed and stored in one of the three types of granules: dense granules, alpha granules, or lysosomes. Dense granules contain mostly small molecules, such as adenosine diphosphate (ADP), ionised calcium, or serotonin (5-HT). Lysosomes encapsulate a variety of hydrolytic enzymes. Alpha granules contain a plethora of cytokines, whose immune functions range from unquestionably pro-inflammatory to serving as indispensable anti-inflammatory agents [23]. Table 1 presents some of the platelet releasates stored in alpha granules. It is unlikely that such a multitude of bioactive substances with contradictory effects is secreted randomly: it is currently believed that platelets are capable of stimulus-dependent packaging and release of granule content.

**Table 1:** Selected platelet releasates stored in alpha granules

Molecule classification	Molecule	Immune function
chemokines	β-thromboglobulin	Chemoattractant and activator for neutrophils
	CXCL4, PF4	Most abundant cytokine in alpha granules; involved in platelet-mediated killing of Plasmodium falciparum; necessary for platelet-induced NETosis; prevents neutrophil apoptosis, activation and adhesion of neutrophils to endothelial cells, phagocytosis and respiratory burst in monocytes, chemotaxis of T lymphocytes; PF4 in the presence of TNF-α induces exocytosis and firm neutrophil interaction with endothelium thereby enhancing inflammation in the lesion; modulates TNF expression; is a potent anti-angiogenic factor by preventing binding of VEGF to endothelial cells
	PPBL, CXCL7	As a result of proteolytic modifications is transformed into connective tissue- and neutrophil-activating peptide, stimulates transendothelial migration of neutrophils
	RANTES, CCL5	Arrests monocyte infiltration of the endothelium
	CCL3, MIP1-alpha	Involved in host defence, targets and induces chemotaxis in multiple immune cells: monocytes, eosinophils, basophils, NK cells, CD8 + T lymphocytes, and DC subsets; significant for development and stabilization of atherosclerotic lesions; induces histamine release from basophils
	CCL-7	Targets and induces chemotaxis in multiple immune cells: monocytes, eosinophils, basophils, NK cells, CD8 + T lymphocytes and DC subsets
Growth factors	VEGF	Involved in physiological angiogenesis and wound healing
	PDGF	Involved in angiogenesis, is potent mitogen for mesenchymal cells including fibroblasts, smooth muscle cells, and glial cells Chemoattracts and activates neutrophils and monocytes; modulates T-cell functions
	TGFβ	Essential permissive factor for metastasis, specifically in epithelial–mesenchymal-like transition
	EGF	Monocyte chemoattractant
	Angiopoietin-1	Preserves vascular integrity at site of inflammation
Cytokines	IL-1β	Activates endothelial and smooth muscle cells; augments neutrophil adhesiveness
	IL-7	Involved in apoptosis resistance, stimulates pro-inflammatory cytokine production
	IL-8	
	HMBG	Activates neutrophils: pericellular granules distribution and release of NETs
	MIP-1-α	See: chemokines
Proteolytic enzymes	Metalloproteinase Inhibitors	
	Protease inhibitors Platelet inhibitor of FXI C1 inhibitor	Attenuates inflammation and promotes resolution, involved in clot dissolution
Other	CD40L, CD154	Induces chemokine secretion and up-regulation of adhesion molecules on endothelial cells; involved in maturation of immunoglobulin affinity and isotype switch in B cells; involved in DC maturation; augmentation of T-cell responses
	PAF	Pro-inflammatory lipid mediator, involved in host defence; activates monocytes, neutrophils and promotes formation of heterotypic platelet aggregates
	Histamine	Pleiotropic immune modulator
	Fibronectin	Involved in host defence
	Vitronectin	Matrix binding protein
	Thrombospondin	Adhesion molecule involved in angiogenesis regulation
	Fibrinogen	Positive loop of platelet activation
	vWF	Positive loop of platelet activation
	HRG	Antimicrobial protein; supresses T-cell activity suppressor, macrophage phagocytosis and formation of immune complexes

Selected molecules stored in platelet alpha granules [6, 21, 23–31]

*CXCL4, PF4* platelet factor 4, *NET* neutrophil-extracellular traps, *TNF* tumor necrosis factor, *VEGF* vascular Endothelial Growth Factor, *PPBL, CXCL7* pro-Platelet Basic Protein, *RANTES, CCL5* Chemokine C–C motif Ligand 5, regulated on activation, normal T-cell expressed and secreted CCL5, *CCL3, MIP1-alpha* Chemokine C–C motif Ligand 3, Macrophage Inflammatory Protein 1-alpha, *NK* natural killer, *DC* Dendritic Cells, *CCL-7* Chemokine Ligand 7, monocyte-chemotactic protein 3, *PDGF* Platelet-derived Growth Factor, *TGFβ* transforming growth factor beta, *EGF* epidermal growth factor, *HMBG* high mobility group box-1, *CD40L, CD154* CD40 Ligand, *PAF* platelet activating factor, *vWF* von Willebrand Factor, *HRG* Histidine-rich glycoprotein

A secreted molecule that deserves particular attention in the context of autoimmunity is CD40L (also known as CD154). It is a membrane glycoprotein expressed by activated platelets [32]. Over a period of minutes to hours, surface-expressed CD40L is cleaved and released in soluble form (sCD40L). It is estimated that over 95% of sCD40L is of platelet origin [33]. Its binding to CD40 on endothelial cells triggers an inflammatory response, such as the release of leukocyte-attracting mediators [34]. It is involved in regulating T-cell function, the activation of dendritic cells, and the regulation of T-dependent antibody isotype switching; it also provides a novel mechanism for platelet autoactivation and the formation of homotypic platelet aggregates [35].

### Protein production de novo

However, platelets are not just storage vesicles that degranulate upon activation. Recent studies revealed that the molecules present in platelets may come from sources other than megakaryocytes. They can be absorbed from the plasma or generated de novo [36]. Surprisingly, these anucleate cytoplasts possess functional spliceosome and transcriptome, accompanied by a range of ribonucleic acids and all the molecular machinery needed to autonomously produce proteins. This was a fascinating discovery, since spliceosome has never been described outside the nuclear boundaries [37]. Spliceosome allows platelets to synthesize proteins de novo in a stimulus-tailored manner. The process was well depicted for a potent pro-inflammatory factor interleukin 1-β (IL1-β) [38]. mRNA for IL1-β is one of the constitutive transcripts in unstimulated platelets [39]. It is located in the polysomes of resting and activated platelets. Accumulation of pro-IL1-β is sustained over hours after platelet activation and followed by processing the precursor into its mature, active form [37]. Integrated post-transcriptional control mechanisms regulate the initiation and resolution of inflammation and give explanation to the role of platelets in immune response and tissue injury [40].

### Platelet microRNA

A fascinating discovery revealed that platelets possess significant amounts of small non-coding RNA, of which around 80% accounts for microRNA (miRNA) [41]. miRNA molecules are thought to post-transcriptionally regulate the expression of over 60% of human genes. The miRNA present in platelets not only influences the functioning of the platelets themselves but also other immune cells, and can both restrain and promote autoimmunity [42]. For instance, miR-146a contributes to controlling the overproduction of cytokines, such as TNF-α, functions as a negative feedback control of innate immunity in TLR signalling and is critical for the suppressor functions of regulatory T lymphocytes. miR-155 promotes the development of pro-inflammatory Th17 and Th1 cell subsets [42].

### Platelet-derived microparticles

Another sign of platelet activation is the generation of microparticles. A range of cells release these small membrane vesicles in both quiescent state and upon activation, but it is platelets that account for over 90% of plasma microparticles found in healthy individuals [9, 43]. Thanks to their size, platelet-derived microparticles (PMP) can easily infiltrate tissues and work as highly efficient carriers of bioactive molecules [44, 45]. They perform this task instead of activated platelets, which acquire binding properties that make it difficult for them to travel through the circulatory system. Microparticles may affect target cells by stimulating them directly via surface-expressed ligands or by transferring surface receptors, which has been reported for both physiological and malignant cells [46]. They modify recipient cell function also by the delivery of cytoplasmic proteins and miRNA [45, 47–49]. They have been shown to increase the expression of adhesion molecules on endothelial and immune cells, enhance cytokine release, and consequently induce angiogenesis [50]. Moreover, PMPs are highly procoagulant and play important role in haemostasis and thrombosis [8, 51]. PMPs provide a surface for complement deposition and participate in complement activation [52, 53]. The fact that circulatory levels of PMPs are significantly elevated in infections, AD, cardiovascular disorders and other pathologic conditions points to their possible role as actual effectors of platelet immune functions [5].

## Platelets in cell–cell interactions

The up-regulation of adhesion molecules and soluble releasates, coherently functioning together, enables platelet–leukocyte interactions [54].

### Platelet–neutrophils

**Fig. 1:**
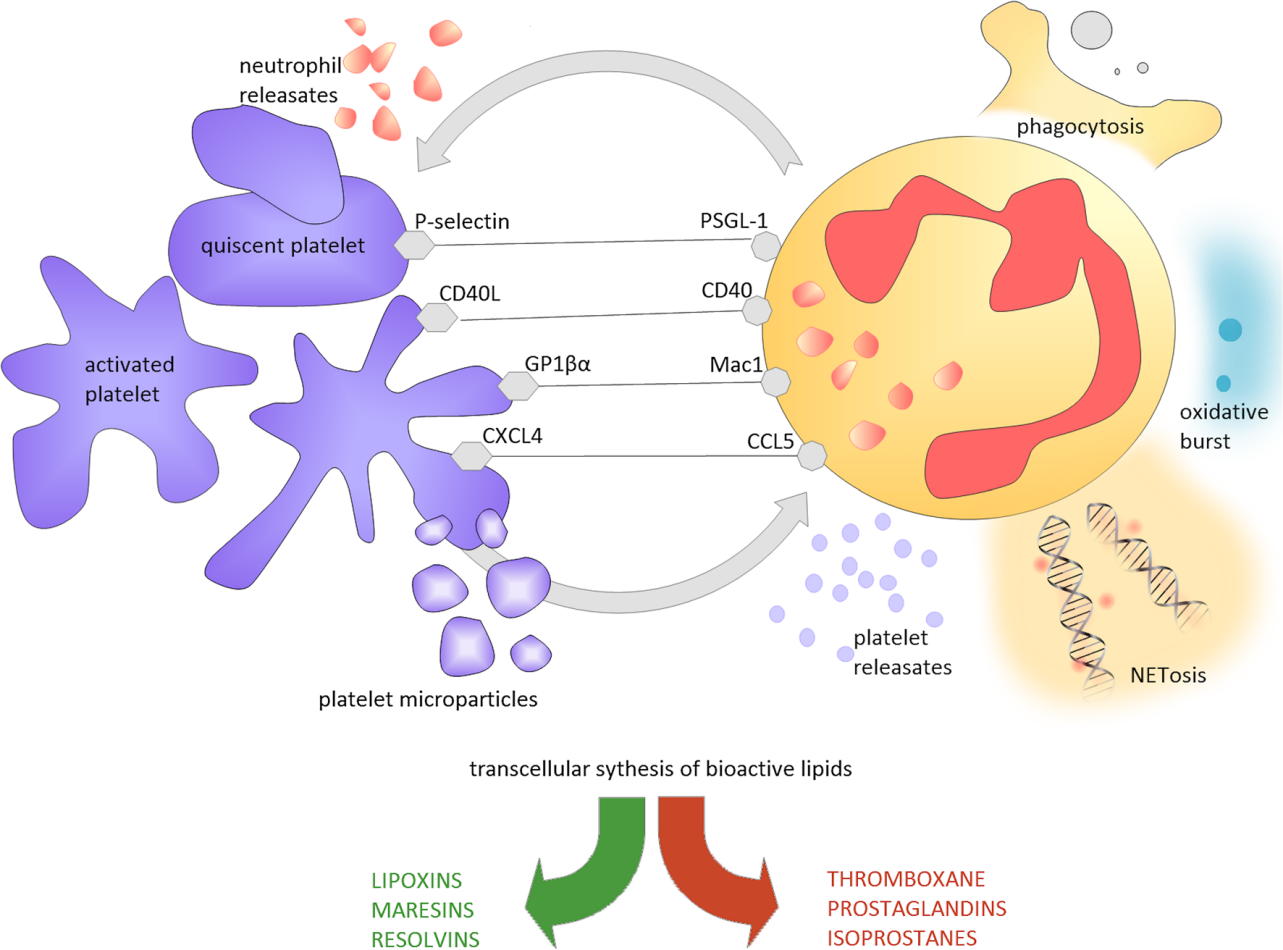
Platelet–neutrophil interactions. The figure presents interactions between platelets and neutrophils

Activated platelets have been shown to adhere to circulating neutrophils and via the release of pro-inflammatory ILβ1, HMGB1, PDGF recruit them to the site of vascular injury [55]. P-selectin expressed on the surface of both activated platelets and endothelial cells promotes the generation of reactive oxygen species, activation of β2 integrins and leukocyte tissue factor, release of pentraxin 3, and proteolytic enzymes [33]. Platelets have an important effect on neutrophils: the induce neutrophil-extracellular trap (NET) formation [56]. This is a form of cell autophagy, distinctive from apoptosis, which is based on the fusion of primary granules with nuclear membrane. It results in the destruction of neutrophil genetic material and its subsequent expulsion to the extracellular environment, where the NET engulfs pathogens. NETs reversely activate platelets, what may lead to a vicious loop of NET formation and platelet activation [57], resulting in tissue injury, prothrombotic phenotype, and propagation of autoimmunity [58]. Interactions with neutrophils also enable platelet extravasation and the targeting of other tissues, as reported in several autoimmune disorders [59]. Platelets support reactive oxygen species generation by monocytes and promote neutrophil oxidative burst and prothrombotic phenotype of monocytes [55, 60]. At the same time, platelet–neutrophil conglomerates enable transcellular synthesis of bioactive lipid compounds, including molecules required for inflammation resolution such as lipoxins, maresins, and resolvins [61, Fig. 1].

### Platelet–lymphocytes

In healthy subjects, about 3% of circulating lymphocytes is bound to platelets. This number increases significantly upon platelet activation and homeostasis deregulation [62, 63]. The proportion varies between lymphocyte subpopulations and depends on the level of platelet activation and the form of stimulus [64]. Larger, activated lymphocytes are more prone to bind to platelets. Surface proteins P-selectin, GPIIb/IIIa, CD40, and CD11b all contribute to the interaction. The presence of the scavenger receptor CD36 on lymphocytes was recognized as proof of interaction with platelets [65].

The co-culturing of autologous platelets and CD4 + T cells enhanced the production of IL-10 and cytokines characteristic for Th1 and Th17 cells: IFNγ, IL-17. Moreover, the T cells were more prone to differentiate towards types Th1 and Th17, which are associated with autoimmunity [66, 67]. IL-17 is an independent predictor of vascular function in rheumatic diseases [68]. At the same time, platelet binding reduces the capability of lymphocytes to proliferate and produce Th1/Th17 cytokines [69]. This suggests that platelets may have a different effect on leukocytes depending on the stage of inflammatory response. Platelets alter immunoglobulin production also through direct contact with lymphocytes B. They are capable of delivering CD40L signalling that is crucial to immunoglobulin affinity maturation and isotype switching and T-cell-dependent humoral immune response [70]. The co-incubation of differentiated B cells with activated platelets was associated with increased in vitro production of IgG1, IgG2, and IgG3 [70]. Moreover, platelets were demonstrated to directly activate naïve T cells by presenting antigens in the context of MHCI and delivering co-stimulatory molecules [71].

### Transcellular synthesis of bioactive lipid mediators

Adhesive platelet–heterotypic cell interactions enable the process of transcellular production of bioactive lipids that act as autacoids [72]. Eicosanoids have gained recognition as regulators of two active, contrary processes: the evolution and resolution of inflammation. The fact that these molecules are unstable and highly bioactive first implied that they must be independently synthesized by different cells. However, only a few cell types are capable of performing the complete biosynthetic process of the limited range of eicosanoids due to lack of key enzymes. Cells must, therefore, share their enzymes and coordinate efforts to produce bioactive lipid compounds in the process of transcellular synthesis [73]. The omnipresence of platelets in blood makes them perfect candidates for this phenomenon. Indeed, platelets are unique cells equipped with a range of enzymes including several forms of phospholipase A2 (PLA2) and 12-lipooxygenase (12-LOX) [9].

Platelets participate in both intensifying inflammation and in its resolution. Upon activation, platelet membrane phospholipid bilayer composition undergoes changes, and substrates are made available for bioactive species production [74]. Platelets can autonomously generate some eicosanoids: thromboxane A2 (TXA2), 12-hydroxyeicosatetraenoic acid (12-HETE), 12-hydroxyheptadecatrienoic acid (12-HHT), prostaglandin E2 (PGE2), prostaglandin D2 (PGD2), and isoprostanes. The molecules have generally proaggregatory and pro-inflammatory properties. Moreover, platelet–neutrophil transcellular biosynthesis of leukotrienes accounts for significant physiological changes associated with an ongoing inflammatory response [75]. Platelets may deliver both arachidonic acid (AA) and leukotriene A4 (LTA4) to neutrophils, as well as other leucocytes and endothelial cells. Prostaglandins have dual role: they initiate inflammatory responses [76], but also induce the transcriptional regulation of neutrophil 15-lipooxygenase (15-LOX). This is the first step in the class switch of lipid mediators [61, 77].

The resolution of inflammation is an active process initiated by a switch in the class of synthesized mediators, from the aforementioned thromboxanes, leukotrienes, and prostaglandins to endogenous pro-resolution lipid mediators: resolvins, protectins, maresins, and lipoxins. Lipoxins may be AA derivatives, but others are derived solely from dietary polyunsaturated fatty acids (PUFAs) [77]. Lipoxins themselves are produced in only three pathways, one of which is performed by platelet–neutrophil aggregates, whereby the neutrophils deliver LTA4, which is consequently transformed into lipoxin B4 (LB4) with platelet 12-LOX. LB4 has potent anti-inflammatory properties: it counter-regulates LT and cytokine production (including TNF), decreases vascular permeability, inhibits neutrophil tissue infiltration, and regulates platelet–neutrophil interactions [61]. These are also affected by ASA-triggered lipoxin (ATL), a molecule synthesized in a pathway enabled by acetylation of cyclooxygenase 2 (COX-2) enzymes [78]. Platelets deliver prostaglandin H2 (PGH2), a COX product, to endothelial cells which transform it into prostacyclin (PGI2). PGI2 works as a counterweight to TXA2. An imbalance between these two molecules may result in the formation of a prothrombotic phenotype [9].

Along with macrophages, monocytes and neutrophils, platelets transform omega-3 essential PUFAs to resolvins, protectins, and maresins. These molecules promote wound healing, deactivate leukotrienes, and attenuate nuclear factor NFkB [79]. They also intensify phagocytosis of apoptotic cells and phagocyte removal via lymphatic vessels. Their anti-inflammatory effect on platelets is based on reducing ADP-dependent platelet aggregation and redirection molecule production onto growth factors and pro-resolving mediators [77]. Platelet–neutrophil aggregates form maresin 1, a compound that is organ-protective [80], and influences blood cells by promoting a pro-resolving platelet phenotype and reposing macrophage functions [81, 82].

## Evidence for platelet contribution in autoimmune disorders

*Rheumatoid arthritis* (RA) is probably the most common connective tissue disease, affecting almost 1% of global population. Cardiovascular disease is the leading cause of death in RA patients [83]. According to European League Against Rheumatism (EULAR) recommendations, the result of traditional cardiovascular risk assessment equations should be multiplied by 1.5 for RA patients [84].

Multiple evidence point towards increased platelet activation in RA patients. Many of them have an increased platelet count. It can be explained by reduced platelet survival and accelerated turnover rate [85]. Intensified platelet production results in higher percentage of young platelets in circulation. Younger platelets are characterized by enlarged size and higher reactivity. Increased amount of soluble platelet releasates, such as: P-selectin, beta-thromboglobulin, or PF4, are found in the serum of RA patients. They were also found to have a higher number of circulating PMPs [86]. Increased platelet activation, assessed with these indicators, correlates positively with disease severity [87–93]. In mouse, model of RA platelet depletion leads to inflammation alleviation [94].

In the serum of RA patients, immune complex and autoantibody characteristics for RA can activate platelets [95]. This includes anti-citrullinated protein antibodies (ACPA) and rheumatoid factor (RF). ACPA–IgE immune complexes may activate platelets via FccRIIA and FceRIa—high- and low-affinity receptor (FceRII/CD23) for IgE, and low-affinity immunoglobulin G (IgG) receptor (FcγRIIa) [17]. The percentage of ACPA to total IgG shows a positive correlation with platelet activation and disease activity [95]. The presence of these antibodies [96, 97] was proposed as a novel risk factor of cardiovascular events [98, 99] and atherosclerotic plaque instability [15, 100, 101].

In vessels as well as in RA-inflamed joints, the exposure to the extracellular matrix stimulates platelets. Platelets bind to exposed collagen via glycoprotein VI membrane receptor (GPVI) and this interaction provokes generation of PMP abundant in IL-1 [102]. Elevated PMP counts were found not only in plasma, but also in the synovial fluid of RA patients. In fact, PMP concentration in synovial fluids was significantly higher than in blood, suggesting that increased PMP generation occurs locally [102].

IL-1, so abundant in synovial fluid PMP, is the key molecule for intercommunication between platelets and fibroblast-like synoviocytes (FLS) [103]. Activated FLS are regarded as key effectors of cartilage destruction [104] and defective angiogenesis in RA [50]. In comparison with healthy subjects, RA patients’ FLS express altered levels of cytokines, chemokines, adhesion molecules, and matrix metalloproteinases, and they also become resistant to apoptosis. Their interaction with platelets expands beyond the soluble cytokines. In the process of transcellular biosynthesis, platelets and FLS produce prostaglandin I2 (PGI2). PMP stimulate up-regulation of eicosanoid synthesis enzymes: COX-2, microsomal prostaglandin E synthase 1 (mPGES-1), and PGE2 [105]. PGE2 itself enhances the expression of membrane-bound mPGE. Independently of PMP generation, and platelet–fibroblast-like synoviocytes aggregate synthesize prostacyclin. The synthesis occurs in a COX-1-dependent manner [106]. Lipid profiling of the synovial fluid of RA patients demonstrated significant amounts of platelet-related mediators, showing disproportion favouring pro-inflammatory lipids, such as LTB4 isomers [107]. In a mouse, model of RA resolution of inflammation is disrupted by inhibition of COX-2, but can be restored by lipoxins [108]. PMP in the synovial fluid of RA patients are highly heterogeneous in size. Larger microparticles were found to contain immune complexes (IC), which, moreover, responded to majority of the IC detected [109]. Immunoglobulins and complement are associated with majority of PMP in RA synovial fluid.

PMP express platelet-derived antigens and associate with autoantigens from other sources that are present in plasma [53]. Platelet-derived antigens include intracellular proteins, such as vimentin. Vimentin externalisation occurs during platelet activation. PMP surface may be a site of further post-transcriptional autoantigen modifications, for example, citrullinisation [110]. This results in the generation of neoepitopes, recognized by autoantibodies characteristic for RA [111]. Complement proteins can bind to PMP surface. Highly bound C1q, C3, and C4 complement components are characteristic for PMP in the synovial fluid of RA patients. These immune complexes were showed to strongly activate neutrophils. Self-sustaining activation of platelets and neutrophils at the site of synovial bleeding may synergize in the formation of citrullinated fibrinogen and vimentin neoepitopes [110]. Proteins of platelet origin are being transformed by peptidylarginine deiminase 4 (PAD4): a neutrophil-derived enzyme [112]. Upheld activation of platelets and neutrophils results in NETosis which, in turn, strongly activates FLS. FLS are capable of internalising NETs. It induces antigen-presenting cell properties in FLS, which can now present platelet-derived, post-transcriptionally modified autoantigens. The process supports autoimmunity [20, 113].

**Fig. 2:**
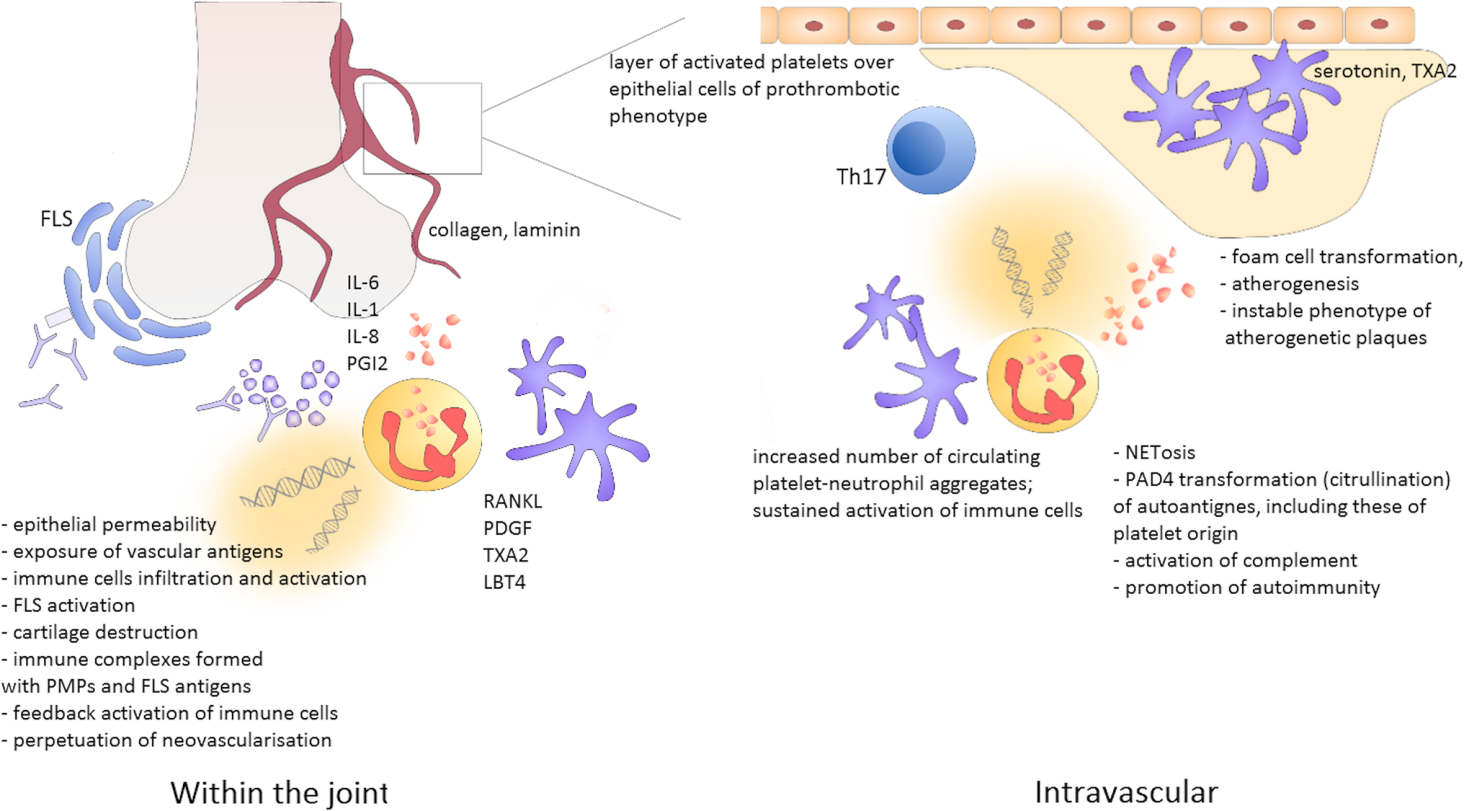
Platelet role in the pathogenesis of rheumatoid arthritis

Finally, platelets contribute to RA synovitis by maintaining persistent permeability of synovial microvasculature. They activate endothelial cells what results in the expression of surface adhesion molecules and enables cell migration [62]. Platelets release serotonin in the spots of vascular damage which cause formation of endothelial gaps with submicron dimension. It favours synovial infiltration by inflammatory cells [114, Fig. 2].

*Systemic Sclerosis* (SSc) is a heterogenous and complex autoimmune disorder with three main components: production of autoantibodies and cell-mediated autoimmunity, vascular damage leading to fibroproliferative vasculopathy and fibroblast dysfunction consisting of excessive accumulation of collagen in organs [115].

Patients suffering from SSc have increased blood levels of platelet activation markers and higher platelet aggregation ratio in comparison with healthy subjects [116–119]. The suggested reason was endothelial dysfunction, considered the primary event in SSc. Subsets of enlarged von Willebrand multimers were found in their plasma, revealing another cause of chronic activation [120]. Platelets become hypersensitive to activating factors such as serotonin, adrenalin, ADP, and collagen. In fact, characteristic overexpression of the non-integrin receptor for collagen I was found in SSc patient platelets. There is growing evidence for existence of a network between platelets and autoreactive T lymphocytes specific against collagen I. Autoreactive T cells produce IFNγ and IL-1, which stimulate megakaryocyte expression of PI3K and Akt. This results in overexpression of these signatures of activation on platelets, enhanced response to collagen I, and platelet aggregation. Exposure to collagen I increases with the disease progression, depicted by gradual fibrosis [120].

5-HT signalling seems to be of particular importance in the activation of platelets in SSc. Concomitant excessive levels of 5-HT in circulation and defective platelet content were found. The presence of a polymorphism in the gene for the serotonin 2A receptor, resulting in weaker platelet activation and aggregation, was associated with reduced susceptibility to SSc and alleviation of the course of the disease [121].

Activated platelets release stored granule contents, and the releasates contribute to imbalance between vasodilators and vasoconstrictors and enhance fibrosis [122]. The mechanisms are well described for lysophospholipids, 5-HT, PDGF, TGFβ, and other growth factors. VEGF stimulates angiogenesis, PDGF activates smooth muscle cells and connective tissue fibroblasts proliferation, and TGFβ may increase collagen and matrix components synthesis. The angiogenesis-promoting factors, however, seem to be inhibited by anti-angiogenic mediators such as PF4, which together with pro-inflammatory and pro-fibrotic agents, contribute to the progression of microvascular damage, defective vascular repair, consequent hypoxia, and fibrosis in patients with SSc [123]. Platelet-derived HMGB1 serum level is higher in SSc patients and associated with microvascular damage [49]. CXCL4, a potent anti-angiogenic chemokine secreted by platelets and dendritic cells, was recognized as parameter predicting disease progression and organ complications [124].

Persistent platelet activation implicates deregulation of platelet–neutrophil interactions [112]. Cell aggregates produce pro-inflammatory lipids, and neutrophils release matrix-degradation enzymes and superoxide radicals. These humoral and cellular components alter the process of reperfusion what results in self-sustaining vasculitis, vascular remodelling, and inflammation [56].

*Systemic lupus Erythematosus* (SLE) In SLE, chronic, systemic inflammation is associated with the formation of autoantibodies and immune complexes, and their subsequent deposition is tissues and eventual organ damage [125]. B cells become hyperactive as a result of escalated stimulation from autoantigens exposed on the surface of apoptotic cells, enhanced by T lymphocytes and other immune cells.

SLE has many haematological manifestations, including thrombocytopenia. Low platelet count is more frequent in SLE patients than increased platelet number [126]. It has been associated with other severe symptoms such as kidney injury, haemolytic anaemia, neuropsychiatric manifestations, and a worse course of the disease [91]. At the same time, the following evident markers of platelet activation are increased in the blood of SLE patients: thromboxane, P-selectin, PMP, and platelet–leukocyte aggregates. Circulating immune complexes, high-affinity antibodies against nuclear constituents, the hallmark of SLE, can activate platelets via the FcγRIIA receptor [87].

Activated platelets contribute to the maturation of dendritic cells (DC) [127]; these are crucial in the pathogenesis of SLE, as their interactions with B- and T cells lead to the production of autoantibodies, including those against nuclear antigens (ANA) and DNA (anti-ds-DNA). PMP-associated CD154 can cause DC activation and soluble CD154 levels were found to be increased in SLE patients and to correlate with disease activity measured by SLE Disease Activity Index. Level of CD154 correlates positively with the presence of anti-phospholipid antibodies in SLE patients [128]. Platelet association with DC promotes the secretion of type I IFN(IFN) [129], which is another important constituent of SLE pathogenesis [130]. In addition, platelets from SLE patients were found to over-express IFN-regulated genes, resulting in up-regulated numbers of the proteins PRKRA, IFITM1, and CD69 leading to increased activation of platelets from SLE patients associated with the type I IFN response [130].

Moreover, platelets themselves and PMP are a source of autoantigens involved in the formation of immune complexes [53]. Platelet autoantibodies were found in SLE patients [131]. The majority of circulating immune complexes formed with microparticles are of platelet origin. PMP may deliver ANA and anti-ds-DNA antibodies, both of which are potent pro-inflammatory agents. Circulating PMP in SLE patients carry increased loads of IgG, IgM, and C1q [132]. IgG PMP are associated with autoantibodies and complement activation, their level correlated with anti-DNA antibodies titres. The platelet surface may also be the site of complement activation and deposition. It has been described as a target for circulating anti-phospholipid antibodies (aPL), with the major antigen recognized by aPL being β2 glycoprotein 1, a member of the complement control protein superfamily [87]. The interaction can occur by mechanisms other than direct recognition of platelet antigens, such as by crosslinking of the apolipoprotein ER2′ receptor, and with the involvement of the FcγRIIA receptor. The complement Cd4 protein specifically accumulates on the platelet surface in SLE patients, correlates with disease activity, and is associated with the presence of anti-phospholipids [20].

A large number of SLE autoantigens is exposed in close proximity to the oxidant-generating site, and this is another way in which platelets may promote autoantigen formation [133]. More frequently, deregulated interactions between platelets and neutrophils occur in SLE [112]: PMP-IC elicit leukotriene production and NET generation by neutrophils [60, 127]. Nuclear autoantigens, including ds-DNA, histones, ANA, and neutrophil cytoplasmic antigens all serve as major component of NETs [131]. NET degradation is impaired in SLE, and patients with defective NET degradation have significantly higher titres of anti-NET and anti-ds-DNA autoantibodies, as well as a higher frequency of developing lupus nephritis. Deregulated platelet–neutrophil interactions result in the creation of a self-sustaining cycle of oxidant generation and necrotic cell formation that stimulates the autoimmune response.

SLE patients have an increased risk of developing cardiovascular disease, stroke, and venous thrombosis, which cannot be explained by classic cardiac risk factors [134]. Platelet activation seems to be a potential link between atherosclerotic lesions and systemic inflammation [20], and many patients have been diagnosed with comorbidity of SLE and APS [8].

*Anti-phospholipid Syndrome* (APS) is a systemic autoimmune disorder directly associated with thrombotic and cardiovascular risk [135]. In addition, anti-phospholipid antibodies are present in around 40% of SLE patients [87], around 20% of RA patients and around 10% of SSc patients [136].

Anti-phospholipid antibodies (APLs), particularly lupus anticoagulant, anticardiolipin antibodies (aCL), and anti-β(2)-glycoprotein I (anti-β2 GPI), are a family of antibodies against phospholipids that can exert pathogenic effects. These antibodies may interfere with the membrane phospholipids of endothelial cells and platelets, or with the bioactive phospholipids of signalling cascades, including the coagulation cascade [137]. Circulating anti-phospholipid antibodies with anti-β2 GPI activity recognize endothelial cells, which synthesize TF4 and express adhesion molecules such as ICAM1 and E-selectin upon recognition. This synthesis results in platelet activation and increased TXA2 production. High levels of TF4 and TXA2 mediate the procoagulant state: a known risk factor of thrombosis.

Thrombocytopenia is commonly observed among APS patients, and the most common determinant autoantigen described is platelet β2-glycoprotein I (β2-GPI). β2-GPI is an apolipoprotein which plays a complex role in blood coagulation. Its active form exposes an epitope recognized by specific IgG autoantibodies. Binding the autoantibodies to β2-GPI results in the formation of a macromolecular complex, PF4-β2-GPI–anti-β2-GPI, that contributes significantly to platelet activation [63]. Platelets may thus be perceived as the primary target of anti-phospholipid antibodies [138], although aCL antibodies can only bind to platelet plasma membrane phospholipids after activation, as the major binding targets are the anionic phospholipids of the inner layer of the membrane. Enhanced platelet activation sustained over time is related to vascular dysfunction and progressive damage, thrombotic events, and complement activation [139]. Immune complexes containing C4d and C3b complement proteins have been found to be deposited in the placentas of APS patients [87]. Activated platelets produce thrombopoietin and contribute to increased platelet turnover, which represents a positive regulatory loop. Despite the augmented production and accelerated maturation of platelets, up to 50% of APS patients develop thrombocytopenia, usually explained as a result of excessive platelet destruction [138].

PMP are significantly increased in APS patients, especially in those with APS secondary to SLE or patients with a history of thrombotic complications [63]. A study of APS thrombus composition revealed a great number of PMP. A higher level of platelet-derived bioactive releasates was also found in APS patients, with most being CXCL4 (PF4) [128]. In APS patients, PF4 interacts with the anti-β2-GPI–β2-GPI macromolecule and further enhances platelet activation. The levels of platelet–leukocyte aggregates are significantly higher in APS patients compared to a healthy population [63].

### Evaluation of platelet activity in clinical practice

The platelet indices such as a mean platelet volume (MPV) and platelet distribution width (PDW) are currently under investigation as possible new indicators of platelet activation. Since they are routinely evaluated as a part of a complete blood count test, they could become a cheap and highly accessible measurement of platelet parameters. The hypothesis is based on the fact that upon activation platelets increase volume and form pseudopodia. Several research teams have attempted to correlate MPV with the RA, SSc, and SLE disease severity and cardiovascular risk; however, they have reported contradictory results [63, 90, 92, 128, 140–142]. In SLE, smaller platelet size was proved to correlate with greater platelet activation, PMP formation, the presence of aCL antibodies, and secondary APS [143]. Changes to MPV have also been investigated in response to the RA treatment with anti-TNFα agent. A recent study showed that platelet count decreased and MPV increased at the end of the treatment period [144]. These discrepancies may be due to a magnitude of variables influencing MPV with age and sex being just the tip of the iceberg. There is an inverse correlation between platelet count and MPV and there are large interindividual differences between both the indices. MPV is also a parameter prone to change upon pre-analytical variabilities [145]. Due to MPV variability, one research team has identified PDW as a more specific marker of platelet activation of coagulation [146]. Even then, there are significant differences in measuring MPV and PDW among blood counters [147]. It remains controversial whether platelet indices correlate with the optical aggregometry results [148].

Another challenge in the interpretation of platelet indices in the systemic AD is the vast prevalence of atherothrombosis among patients. MPV is a well-established predictor of cardiovascular risk [149]. Increased MPV was associated with an acute myocardial infarction, ischemic stroke, and atrial fibrillation [150]. High MPV predicts a poor outcome of these events [151]. As rheumatic disorders are known to increase the risk of atherosclerosis and its complications, it cannot be excluded that increased MPV in AD reflects the existing subclinical atherosclerosis. Therefore, altered MPV value in AD patients could be an expression of an overlapping cardiovascular disease. At the same time, both systemic AD and chronic inflammatory diseases (including cardiovascular disease) are characterized by a high level of IL6. IL6 may stimulate the bone marrow to an increased release of younger, bigger platelets [152].

Future studies could investigate these issues further by designing research protocols including several different methods of platelet reactivity assessment. This includes well documented methods such as light transmission aggregometry, flow cytometry, and quantification of platelet-derived soluble proteins in blood plasma.

## Conclusions

Platelets recently became an intriguing topic of research on mechanisms of autoimmunity. Growing evidence on platelet role in autoimmune disorders is being delivered by independent research teams.

There is no doubt that platelets are a source of autoantigens. Their prolonged activation may lead to the recognition of surface molecules by autoantibodies, or to their enzymatic transformation and the formation of neoepitopes. In autoimmune-mediated inflammation, platelets act as a component of the positive feedback loop response by secreting and synthesising vast amounts of bioactive compounds. They contribute to pathological hallmarks of autoimmune inflammation such as: extensive complement activation, circulation of immune complexes, impaired phagocytosis of apoptotic cells, and NETs. Platelets promote T helper leukocyte maturation towards type 1 and type 17, associated with autoimmunity. Costimulation signal delivered by platelets enable maturation of antibodies affinity. The enzymes and growth factors delivered by platelets facilitate tissue degeneration and neovasculogenesis. Mechanisms and pathways described for platelets in the context of AD overlap with those characteristic of thrombosis and atherosclerosis, which explains the increased cardiovascular risk of AD patients [5, 8, 88, 153].

However, platelets also play an opposite role. They are indispensable in the production of pro-resolution and anti-inflammatory lipid mediators, which trigger adaptive immunity, promoting a shift in balance between types of Th lymphocytes and dendritic cells. These compounds enhance the clearing of apoptotic cells and immune complexes—potential autoantigens. Platelet-rich plasma (PRP) infusions are widely applied in treating joint trauma, as platelet-derived growth factors have a positive influence on chondrocytes [154, 155]. A recent study proposes an immunological mechanism of resolving antigen-induced arthritis and implementation of PRP infusions in rheumatology. In a porcine model of RA, PRP was found to alleviate disease symptoms [156]. Platelets can internalize larger IgG-coated particles in a process similar in some respects to phagocytosis in leukocytes, thus supporting the clearance of immune complexes. Finally, platelet binding to Th lymphocytes reduce their ability to produce cytokines typical for autoimmunity-related subtypes.

The evidence for platelet involvement in the patomechanisms of autoimmune disorders suggests that platelets play either a pro- or anti-inflammatory role depending on the inflammation-initiating stimuli [28, 157, 158]. The main limitation of the most of the available studies is that they only present a selected phase of platelet activity. Furthermore, they often use a single marker to assess it. It would be important for the future research to present platelet activity as a dynamic, variable process. A further investigation of the platelet traditional indices (like MPV and PDW) and the other markers (soluble platelet-derived protein assays, flow cytometry, PMP, and optical aggregometry) could provide an attractive tool for clinicians. It would be worth to investigate the influence of the available anti-rheumatic medication on platelet function. Other examples of the potential areas for further research include, for instance, the role of platelet-derived miRNA in AD or PMP as carriers of bioactive molecules. The authors hope that the exploration of the role of platelets as a potential therapeutic target and a possible ally in the treatment of autoimmune disorders will continue.
